# Facts and Gaps in Exercise Influence on Arrhythmogenic Cardiomyopathy: New Insights From a Meta-Analysis Approach

**DOI:** 10.3389/fcvm.2021.702560

**Published:** 2021-10-18

**Authors:** Julia Martínez-Solé, María Sabater-Molina, Aitana Braza-Boïls, Juan J. Santos-Mateo, Pilar Molina, Luis Martínez-Dolz, Juan R. Gimeno, Esther Zorio

**Affiliations:** ^1^Cardiology Department, Hospital Universitario y Politécnico La Fe, Valencia, Spain; ^2^Laboratorio de Cardiogenética, Unidad de Cardiopatías Familiares, Instituto Murciano de Investigación Biosanitaria (IMIB-Arrixaca), Murcia, Spain; ^3^Unidad CSUR (Centros, Servicios y Unidades de Referencia) en Cardiopatías Familiares, Hospital Universitario Virgen de la Arrixaca, Murcia, Spain; ^4^CIBERCV, Center for Biomedical Network Research on Cardiovascular Diseases, Madrid, Spain; ^5^Unidad de Cardiopatías Familiares, Muerte Súbita y Mecanismos de Enfermedad (CaFaMuSMe), Instituto de Investigación Sanitaria La Fe, Valencia, Spain; ^6^Cardiology Department, Hospital Universitario Virgen de la Arrixaca, Murcia, Spain; ^7^Instituto de Medicina Legal y Ciencias Forenses de Valencia, Histology Unit, Universitat de València, Valencia, Spain

**Keywords:** sports, exercise, arrhythmogenic cardiomyopathy, disease progression, risk factors

## Abstract

Arrhythmogenic cardiomyopathy (ACM) is a genetic cardiac condition characterized by fibrofatty myocardial replacement, either at the right ventricle, at the left ventricle, or with biventricular involvement. Ventricular arrhythmias and heart failure represent its main clinical features. Exercise benefits on mental and physical health are worldwide recognized. However, patients with ACM appear to be an exception. A thorough review of the literature was performed in PubMed searching for original papers with the terms “ARVC AND sports/exercise” and “sudden cardiac death AND sports/exercise.” Additional papers were then identified through other sources and incorporated to the list. All of them had to be based on animal models or clinical series. Information was structured in a regular format, although some data were not available in some papers. A total of 34 papers were selected and processed regarding sports-related sudden cardiac death, pre-clinical models of ACM and sport, and clinical series of ACM patients engaged in sports activities. Eligible papers were identified to obtain pooled data in order to build representative figures showing the global incidence of the most important causes of sudden cardiac death in sports and the global estimates of life-threatening arrhythmic events in ACM patients engaged in sports. Tables and figures illustrate their major characteristics. The scarce points of controversy were discussed in the text. Fundamental concepts were summarized in three main issues: sports may accelerate ACM phenotype with either structural and/or arrhythmic features, restriction may soften the progression, and these rules also apply to phenotype-negative mutation carriers. Additionally, remaining gaps in the current knowledge were also highlighted, namely, the applicability of those fundamental concepts to non-classical ACM phenotypes since left dominant ACM or non-plakophillin-2 genotypes were absent or very poorly represented in the available studies. Hopefully, future research endeavors will provide solid evidence about the safest exercise dose for each patient from a personalized medicine perspective, taking into account a big batch of genetic, epigenetic, and epidemiological variables, for instance, in order to assist clinicians to provide a final tailored recommendation.

## Introduction

**Figure 1:**
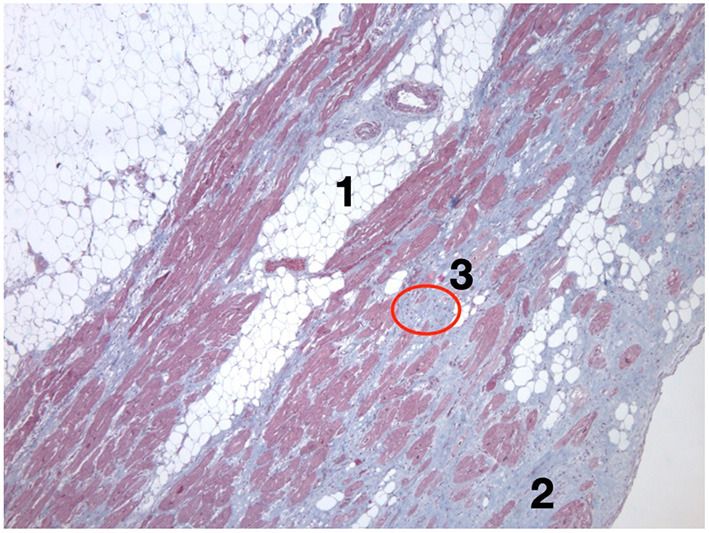
Histological view of the left ventricle of a heart with biventricular arrhythmogenic cardiomyopathy due to the TMEM43 S358L mutation. This patient suffered a sudden cardiac death while practicing sports. The histological hallmarks that define the disease are shown, such as myocardial loss due to fatty infiltration (1) and fibrosis (2); additionally, lymphocytic infiltrates can be observed (3). TMEM43, transmembrane protein 43.

The classical definition of arrhythmogenic cardiomyopathy (ACM) refers to a rare genetic disease resulting in myocardial loss and fibrofatty substitution of the ventricular myocardium, involving either right, left, or both ventricles (1, 2) and often presenting inflammatory infiltrates (1) (Figure 1). However, in the last years a broader definition of the disease has been proposed to include under this umbrella term also other acquired and genetic pathological entities which share a primary myocardial involvement and a clinical presentation with arrhythmias such as myocarditis, sarcoidosis, amyloidosis, sarcomeric, and mitochondrial defects (3). From now onward, this review is focused only on the original definition of ACM whose reported incidence is 1:5,000 in general population (3).

The genetic basis of ACM has been widely expanded since the initial identification of mutations in the genes encoding desmosomal proteins, and currently other disease-causing non-desmosomal genes have also been recognized (1). Mutations in some genes are prone to present with classical forms of the disease and profound structural and electrical alterations arising from the right ventricle while mutations in other genes tend to involve the left ventricle either in isolation or as the main feature of the disease (Table 1) (1, 3–7). Mutations can be identified in 50% of the probands, and the family screening often confirms an incomplete penetrance and a variable expression of the disease. Thus, other factors are thought to play a relevant role as modulators to explain these clinical findings, including epigenetics, virus, sexual hormones, and sports (3). Importantly, gene elusive patients should not be reassessed as having a non-genetic disease since not all ACM genes are known so far and/or certain types of mutation might not be detected with the technology routinely employed (i.e., big rearrangements could be missed by conventional NGS sequencing without copy number variation analyses).

**Table 1:** List of arrhythmogenic cardiomyopathy-causing genes and the main characteristics of their phenotype.

**Gene name**	**Gene symbol**	**Gene location**	**RV involvement**	**Biventricularinvolvement**	**LV involvement**	% In probands^*^
Plakoglobin	JUP	17q21.2	Yes	Yes	No	0–1
Desmoplakin	DSP	6p24.3	No	Yes	Yes	3–15
Plakophillin-2	PKP2	12p11.21	Yes	Yes	No	20–46
Desmoglein-2	DSG2	18q12.1	Yes	Yes	Yes	3–20
Desmocollin-2	DSC2	18q12.1	Yes	Yes	No	1–8
Transforming growth factor beta-3	TGFB3	14q24.3	Yes	No	No	?
Transmembrane protein 43	TMEM43	3p25.1	Yes	Yes	No	0–2
Titin	TTN	2q31.2	Yes	Yes	Yes	0–10
Desmin	DES	2q35	No	Yes	Yes	0–2
Filamin C	FLNC	7q32.1	No	No	Yes	3
Lamin A/C	LMNA	1q22	No	Yes	Yes	0–4
Phospholamban	PLN	6q22.31	No	Yes	Yes	0–1
Alpha-3 catenin	CTNNA3	10q21.3	Yes	Yes	No	0–2
Cadherin-2	CDH2	18q12.1	Yes	Yes	No	0–2
Sodium voltage-gated channel, alpha subunit 5	SCN5A	3p22.2	Yes	Yes	Yes	2
RNA-binding motiv protein 20	RBM20	10q25.2	No	No	Yes	1
LIM domain binding 3	LDB3	10q23.2	Yes	No	No	?
Tight junction protein 1	TJP1	15q13.1	Yes	Yes	No	<5%

* *Except in specific geographical regions, where it could be much higher. Modified from (1, 3–7). ?: unknown*.

Mutations in ACM mostly involve desmosomal genes and affect the composition of the intercalated disk. Structural remodeling at the intercalated disks yields subsequent electrical remodeling at the neighboring gap junctions and sodium channels and, furthermore, modifications in nuclear signaling and transcriptional activity mediated by Wnt and Hippo pathways (8, 9). The final myocardial substitution by fibrofatty tissue (Figure 1) provides the macroscopic anatomical substrate for the ventricular arrhythmias that characterize the disease, but also, at a subcellular scenario, the abovementioned gap junction and sodium current remodeling promote patchy slow conduction areas and re-entrant circuits for ventricular arrhythmias (3).

Exercise and sports practice confer beneficial effects on such a wide variety of organs that their recommendation in general population remains out of debate (10). Despite this compelling evidence, in the last years extensive data have been published to support an adverse influence on patients with ACM. Thus, clinical guidelines have accordingly been released to restrict sports recommendations in this population (Table 2) (11), yet a low-intensity exercise is recommended to these patients. A practical advice could be to avoid exercise levels that hamper maintaining a conversation (13).

**Table 2:** Modified exercise recommendations for patients with arrhythmogenic cardiomyopathy included in the European Society of Cardiology guidelines.

**ESC 2020 exercise recommendation**	**Class of recommendation**	**Level of evidence**
Participation in 150 min of low-intensity exercise per week should be considered for all individuals.	IIa	C
Participation in low- to moderate-intensity recreational exercise/sports^*^, if desired, may be considered for individuals with no history of cardiac arrest/VA, unexplained syncope, minimal structural cardiac abnormalities, <500 PVCs/24 h, and no evidence of exercise-induced VAs.^*^This category includes bowling, cricket, curling, golf, riflery, and yoga.	IIb	C
Participation in high-intensity recreational exercise/sports or any competitive sports is not recommended in individuals with ACM, including those who are gene positive but phenotype negative.	III	B

*Low-intensity, light exercise: below the aerobic threshold, <40% of maximum oxygen consumption, <55% of maximum heart rate, <40% of heart rate reserve, rate of perceived exertion scale 10-11. Moderate-intensity exercise: between the aerobic and the anaerobic thresholds, 40–69% of maximum oxygen consumption, 55–74% of maximum heart rate, 40–69% of heart rate reserve, rate of perceived exertion scale 12-13. VA, ventricular arrhythmias; PVC, pre-mature ventricular contraction; ACM, arrhythmogenic cardiomyopathy (11, 12)*.

Exercise testing is usually included in the routine study protocol triggered by the suspicion of ACM ever since it was recommended in the original Task Force criteria for probands and family members. These consensus documents considered left bundle branch ventricular arrhythmias recorded at different tests (including exercise testing) as a minor criterion for probands and as one of the four additional criteria besides being a first-degree relative for family members (14). A challenging paper demonstrated that exercise testing could unmask depolarization and repolarization abnormalities as well as ventricular arrhythmia in mutation carriers, irrespective of the fulfillment of Task Force criteria and the symptomatic state, suggesting a potential use of this test to prescribe exercise in case it could be validated in that scenario (15). Against this hypothesis, another interesting work compared the arrhythmogenicity response during the isoproterenol and the exercise test in 37 ACM patients and underlined the frequent and falsely reassuring reduction or abolishment of baseline ventricular arrhythmia during the exercise testing (16). Only one paper has assessed the safety and usefulness of cardiopulmonary exercise testing in 38 ACM patients, concluding that it is safe and that the ventilatory efficiency may predict heart transplantation-free survival (17). However, no studies have been carried out focused on the role of exercise and cardiopulmonary exercise testing to prescribe exercise in ACM patients engaged in sports and followed during a suitable period of time. Additionally, one may acknowledge that it is hard to extrapolate the ideal timely situation of these tests performed in a hospital setting with a random training session in which hydration, blood volume, electrolytes, acid–base balance, and catecholamine levels can widely vary and transiently increase the baseline electrical instability in ACM patients necessary to trigger ventricular arrhythmias during sports. Thus, no specific recommendation can be given regarding this issue and the authors strongly advise to adhere the current guidelines (Table 2) designed on the exercise dose without considering the result of any type of exercise testing and based on the body of evidence discussed in the following sections.

From a mechanistic point of view, the pressure overload produced by physical exertion may cause a stronger wall stress and more severe myocardial damage at the right ventricle than at the left ventricle (3, 13, 18). Thus, as long as no other factor played any other role, a more deleterious effect on ACM with right ventricular involvement might be expected than in left dominant forms. Damage may include abnormal signaling promoting apoptosis, fibrosis, and adipogenic and inflammatory cascades (13). The last one might be exaggerated in trained patients with ACM or mutation carriers since prolonged periods of intensive physical training can additionally depress immunity and promote inflammation as well (19).

Herein we present a review of the published evidence regarding exercise and sports practice in ACM patients, highlighting the remaining gaps to be addressed in the future.

## Methods

**Figure 2:**
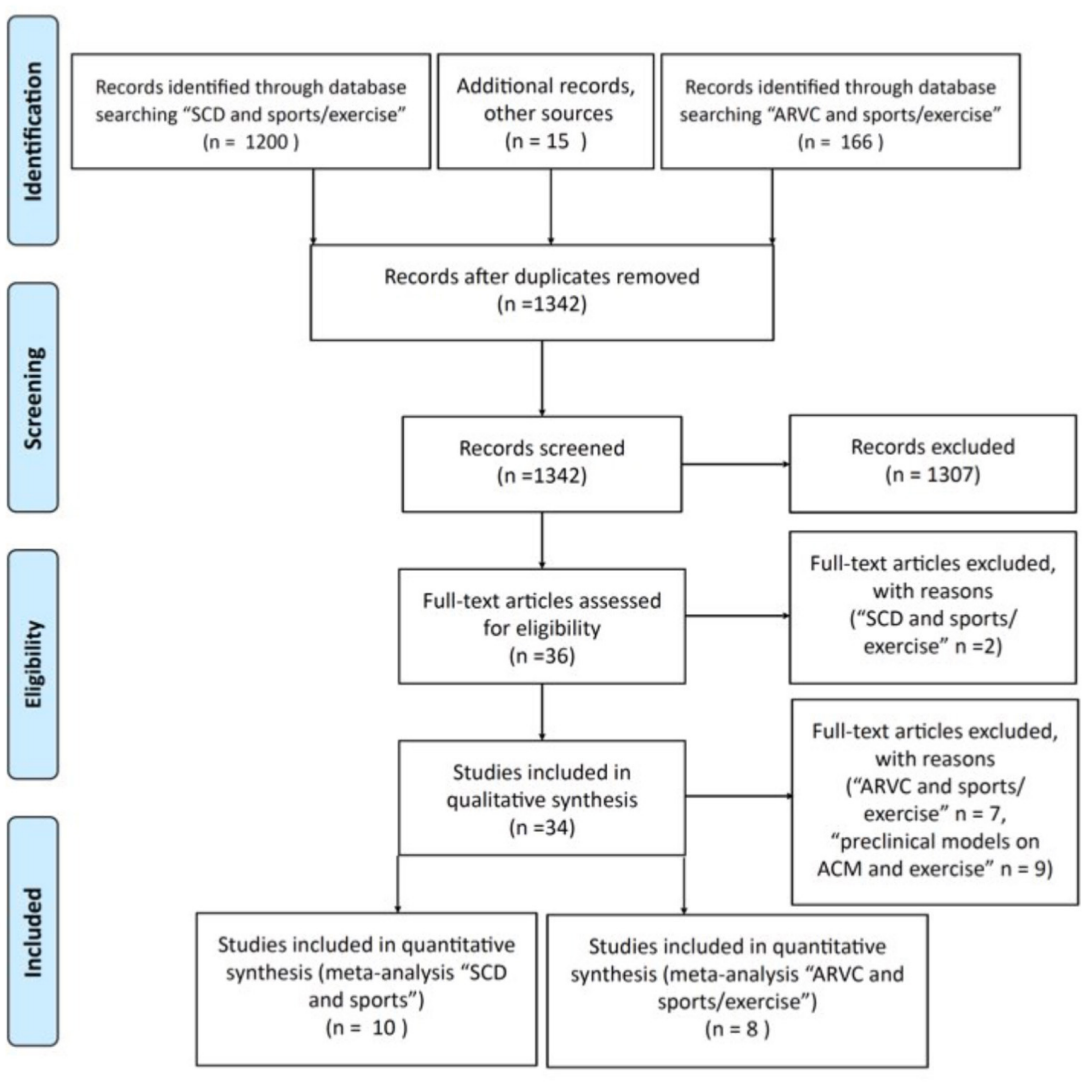
PRISMA flowchart for the identification of papers to obtained pooled data on the causes of sports-related sudden cardiac deaths and on the estimates of life-threatening arrhythmic events in ACM patients engaged in sports. SCD, sudden cardiac death; ARVC, arrhythmogenic cardiomyopathy.

A thorough review of the literature was performed in PubMed by searching papers with the terms “sudden cardiac death (SCD) AND sports/exercise” and “ARVC AND sports/exercise” between 2002 and February 2021 and in English language (Figure 2). This search yielded 1,366 papers, and 15 additional papers were then identified through other searches and/or cited by reviews. Duplicates were eliminated and records screened yielding 36 original papers assessed for eligibility. The information given in these papers was structured in a regular format and presented in Tables, although some data were not available in some papers. We next excluded two papers from the search “SCD AND sports/exercise” because data from SCD cases could not be differentiated from those from cases surviving from sudden cardiac arrests (*n* = 1) and because the same research group had published another selected paper with a bigger sample size which included the excluded paper (*n* = 1). At this point of the PRISMA flowchart, some other studies were excluded for quantitative analyses because relevant numerical data were missing but nonetheless were considered suitable for full-text qualitative synthesis and structured in descriptive Tables dealing with preclinical models on ACM and exercise (*n* = 9) and “ARVC AND sports/exercise” (*n* = 7). The final quantitative synthesis to analyze the global prevalence of the different causes of sports-related SCD and the quantitative synthesis to estimate the life-threatening arrhythmic events (LAEs) in ACM athletes were performed as follows. Proportions for each diagnosis as the cause of death in sports-related SCD series were extracted from each of the 10 selected studies, and their 95% confidence intervals (CIs) were calculated. Annual LAE rates of ACM athletes were calculated for each of the eight selected studies, and CIs were calculated by using the Poisson distribution. Standardized (5-year) rates of LAE were presented in forest plot graphs, and odds ratio calculations were obtained with a meta-analysis dedicated software [Review Manager (RevMan) 5.4. Copenhagen: The Nordic Cochrane Center, The Cochrane Collaboration, 2020]. Three main sections were prepared, namely, observational studies regarding ACM in sports-triggered sudden cardiac death, preclinical ACM models, and exercise and clinical series analyzing the effect of exercise on ACM. Finally, an additional fourth section supporting the possibility of strenuous exercise as stand-alone cause of ACM was added to the present manuscript.

## Observational Studies: ACM in Sports-Triggered Sudden Cardiac Death

The causes underlying a heart arrest or an SCD of someone practicing sports have focused the attention of researchers. Published papers have tried to shed some light on the causes of sports-related SCD from a wide range of settings and based on a variable percentage of forensic studies. They have underlined that ACM represents a not negligible cause of death in this scenario, accounting for roughly 0–28% of the autopsied cases (20–29). The fact that in non-autopsy-based studies ACM is sometimes not even mentioned highlights the difficulties in getting a firm diagnosis when cardiac pathology is missing, as it often happens both in SCD and in sudden cardiac arrest reports (29, 30). Moreover, the pathological overlap of ACM, myocarditis, and idiopathic left ventricular fibrosis allows to speculate that some of the cases with those diagnoses should further increase the real percentage of ACM as the cause of death in case a molecular autopsy confirmed this hypothesis. Supplementary Table 1 summarizes the main papers reporting the cause of death in sports-related SCD series. As expected, the percentage of SCD attributable to coronary artery disease (CAD) increases paralleling the age of the recruited victims, with a maximal prevalence (63%) in the Spanish study by Morentin et al. (*n* = 288) which also reported male predominance and the highest mean age (44 ± 14 years old) of the reviewed series (28). For unknown reasons, even though Spain is considered a country with low prevalence of CAD, its 63% CAD prevalence (28) is double as much as that observed in the Danish series reported by Risgaard et al., with a similar age (41 ± 10 years old) and also with male predominance but with a remarkably smaller sample size (*N* = 44).

**Figure 3:**
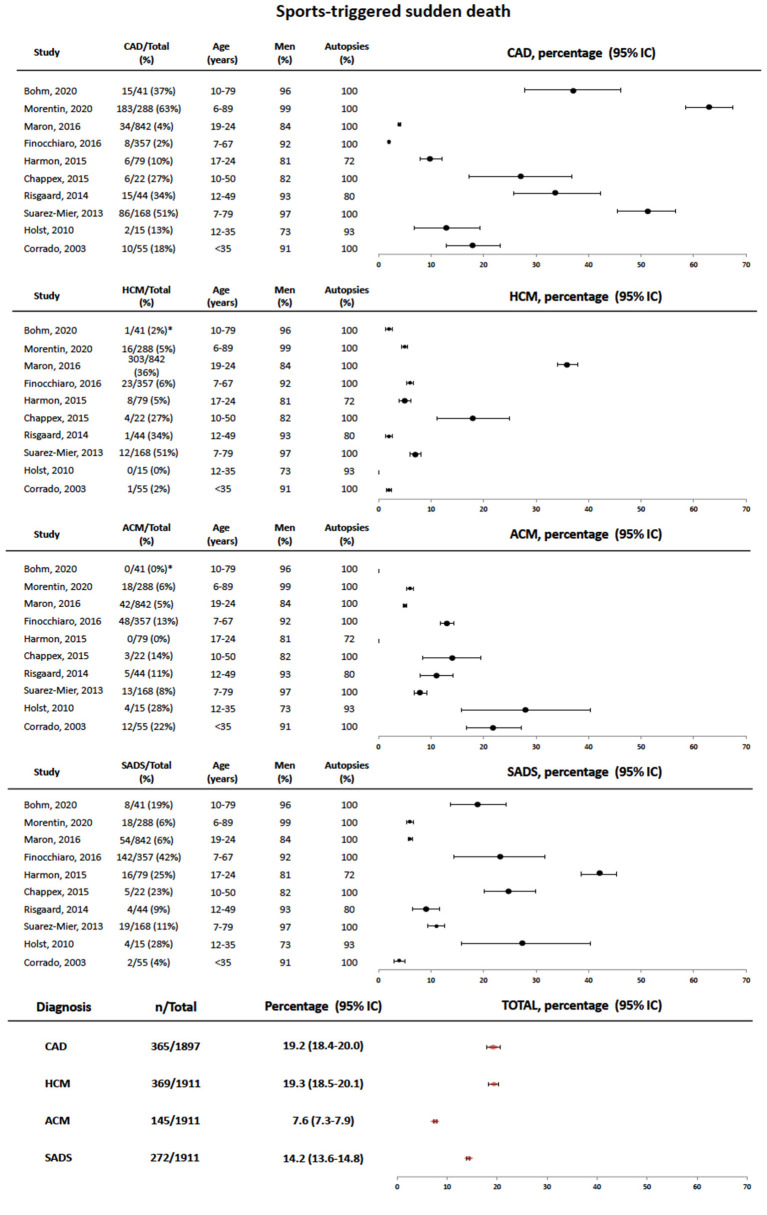
Forest plot showing pooled data of the causes of sports-triggered sudden cardiac death in different series referenced in the text. At the bottom, total estimates are provided for each diagnosis. SCD, sudden cardiac death; CAD, coronary artery disease; SADS, sudden arrhythmic death syndrome; HCM, hypertrophic cardiomyopathy; ACM, arrhythmogenic cardiomyopathy. *The causes of death can be only retrieved form the 41 cases of sports-related sudden death cases with autopsy.

Pooled data obtained from papers in Supplementary Table 1 are shown in Figure 3 with the global estimates of the prevalence of CAD, hypertrophic cardiomyopathy, ACM, and sudden arrhythmic death syndrome in these series. Our data show that CAD and hypertrophic cardiomyopathy should be considered the leader causes of sports-related SCD, although in the youngest subsets of victims inherited cardiac conditions such as hypertrophic cardiomyopathy, ACM, and sudden arrhythmic death syndrome clearly prevail (20, 21, 24, 27). Right after CAD and hypertrophic cardiomyopathy, sudden arrhythmic death syndrome represents the third global cause of death in sports-related SCD. As cardiogenetic knowledge and resources continue to grow, more molecular autopsies will be hopefully performed and thus specifically ascribe these deaths to certain syndromes such as long QT syndrome, Brugada syndrome, and catecholaminergic polymorphic ventricular tachycardia. Taking into account the prevalence in general population of hypertrophic cardiomyopathy and ACM (1:500 vs. 1:5,000, respectively) and our global estimates for them in sports-related SCD series (roughly 20 and 10%, respectively), another important remark is that the risk for developing a sports-related SCD in ACM patients is five-fold that of patients with hypertrophic cardiomyopathy. In keeping with these data, in 72% of 66 ACM cases recruited after a sudden cardiac death or a sudden cardiac arrest, events occurred on exertion (31). Although the genetic background of the geographical area in which the studies were performed and the implementation of pre-participation screening may have influenced the final outcomes, it appears clear that ACM is overrepresented in sports-triggered sudden cardiac death.

## Preclinical ACM Models and Exercise

Animal models provide a useful setting to test hypotheses that cannot easily be tested in *in vitro* cultures, such as the global effect of exercise in an organism in terms of development of both arrhythmic and structural burden. Table 3 updates the major findings of the effect of exercise in several animal models of ACM. Additionally, a paper based on cell cultures assuming stress shear as a subrogate of exercise has been incorporated.

**Table 3:** Pre-clinical ACM models to test the effect of exercise on the phenotype.

	**Type of model**	**Age of the mice**	**Exercise protocol details**	**Results**
Kirchhof et al. (32)	Heterozygous plakoglobin-deficient mice (PG–/+) vs. WT	10 months old	Swimming endurance protocol, 8 weeks	PG–/+ mice exhibited structural and arrhythmic ACM features. Isolated, perfused PG–/+ hearts had spontaneous VT of RV origin and prolonged RV conduction times compared with WT hearts. Endurance training accelerated abnormalities in PG–/+ mice. RV histology and electron microscopy were normal in affected animals.
Fabritz et al. (33)	Heterozygous plakoglobin-deficient mice (PG–/+) vs. WT	10 months old	Swimming endurance protocol, 7 weeks Mice were randomized to a load-reducing therapy (furosemide and nitrates) or placebo	Therapy prevented training-induced RV enlargement in PG–/+ mice. Untreated PG–/+ hearts had reduced RV longitudinal conduction velocity, more spontaneous macro re-entrant VTs than treated and WT and lower concentration of phosphorylated C × 43 than WT, especially in those with VTs. PG–/+ hearts showed reduced myocardial plakoglobin concentration, whereas b-catenin and N-cadherin concentration was not changed.
Lyon et al. (34)	Homozygous desmoplakin-deficient mice (DSP–/–) vs. WT	4 months old	Running on a treadmill, 5 days of acclimatization and 1 session of at least 45 min or until exhaustion, then, mice received a high (2 mg/kg) or low (0.5 mg/kg) epinephrine dose	Increased PVCs were observed in DSP–/– mice when exposed to exercise and epinephrine. Interestingly, high doses of epinephrine in combination with exercise could also induce sudden cardiac death in DSP–/– mice that was not observed in littermate controls.
Hariharan et al. (35)	Cell cultures expressing mutant plakoglobin (JUP) or plakophillin-2 (PKP2) vs. knockdown cell cultures vs. WT cell cultures	-	Cell–cell adhesion assays, immunoblotting, atomic force microscopy, immunofluorescence, TUNEL assay and shear flow experiments (to simulate strenuous exercise) were performed	Mutant cells showed no differences, while knockdown cultures showed weakened cell–cell adhesions. Upon shear stress, mutant cultures failed to increase the amount of immunoreactive signal for plakoglobin or N-cadherin, as observed in WT. In contrast to WT, apoptosis was increased in cells expressing mutant JUP, both in resting conditions and also in response to shear stress. Abnormal responses to shear stress associated with mutant JUP or PKP2 could be reversed by SB216763 (SB2), a GSK3b inhibitor.
Cruz et al. (36)	Heterozygous PKP2 R735X mice (PKP2–/+), obtained with AAV9 technology	AAV9 injection at 4–6 weeks oldExercise 2 weeks later	Swimming endurance protocol, 8 weeks	CMR at 10-months post-infection detected an overt RV ACM phenotype and histologically C × 43 dyslocalization in trained PKP2–/+ mice but not in their sedentary littermates.
Martherus et al. (37)	Heterozygous mice for the human desmoplakin gene carrying DSP R2834H vs. WT	4 months old	Running on a treadmill, 12 weeks	DSP R2834H mice displayed structural features of RV ACM with normal LV and endurance exercise accelerated ACM pathogenesis paralleling a perturbed AKT1 and GSK3-β signaling pathways.
Chelko et al. (38)	Homozygous DSG2 and heterozygous JUP mutant mice vs. WT	3 months old.	Graded exercise training (swimming) since week 5 until 12. Since week 3 some mice started treatment with SB216763 (SB2)	SB2 prevents myocyte injury and cardiac dysfunction *in vivo* in two murine models of ACM at baseline and in response to exercise (in terms of LVEF, survival, and normalization of intercalated disk distribution or in terms of ventricular ectopy and myocardial fibrosis/inflammation reduction, respectively).
van Opbergen (39)	Heterozygous plakophillin-2-deficient mice (PKP2–/+) vs. WT	4 months old	Running on a treadmill, 4 weeks	In PKP2–/+ mice, protein levels of Ca2+ -handling proteins were reduced compared to WT. Trained PKP2–/+ showed a pro-arrhythmic remodeling with RV lateral connexin43 expression, RV conduction slowing, and a higher susceptibility toward arrhythmias.
Cheedipudi (40)	Heterozygous desmoplakin-deficient mice (DSP–/+) vs. WT	6 months old	Running in a treadmill, 12 weeks	A differential gene expression was observed in DSP–/+ vs. WT mice, including upregulated genes inhibitors of the canonical Wnt pathway. Exercise restored transcript levels of 2/3rd of the differentially expressed genes. The changes were associated with reduced myocardial apoptosis and eccentric cardiac hypertrophy without changes in cardiac function and arrhythmias.

*WT, wild type; Cx43, Connexin43; GSK3-β, glycogen synthase kinase 3-β; AKT1, protein kinase B; LV, left ventricular; RV, right ventricular; CMR, cardiac magnetic resonance imaging*.

In summary, trained heterozygous transgenic mice suffered adverse cardiac remodeling. All in all, experiments with plakoglobin, plakophillin-2, and desmoplakin transgenic animals revealed a pro-arrhythmic remodeling with an impaired cardiac electrical conduction and an altered expression of Ca2+-handling-related proteins, with controversial results with respect to development of structural abnormalities in this scenario, as summarized in Table 3. The great differences in the selected genetic background, the study protocols, and their endpoints foreclose precise comparisons between these studies and/or data processing to obtain pooled data with meta-analysis strategies. Slowed conduction was probably the consequence of the C×43 dyslocalization and reduced sodium current (33, 36) and the substrate for ventricular arrhythmias and increased inducibility (32–34, 39). Additionally, exercise-triggered development of fibrosis, apoptosis, chamber enlargement, and systolic dysfunction was reported by some authors (32, 33, 36–38) but not by others (39). Interestingly, pretreatment with preload-reducing therapy (furosemide and nitrates) softened the ACM phenotype after exercise, in terms of both structural and electrical abnormalities (33). Moreover, similar results to these were observed with intervention to downregulate the canonical Wnt pathway by inhibiting GSK3b with SB216763, either in sedentary and exercised mutant mice (38) or in cell cultures exposed to shear stress (35). Thus, in keeping with the current pathophysiology of ACM, down-expression of the Wnt pathway does play a key role in ACM development under exercise and wall stress conditions, which challenges the desmosome integrity and the electrical stability. In contrast, only one paper supported a somehow beneficial effect of exercise on ACM. Indeed, it showed a restoration of the abnormal baseline expression pattern in the left ventricular myocardium of DSP-mutant mice upon exercise, so that it reduced myocardial apoptosis and induced eccentric cardiac hypertrophy without affecting cardiac function or arrhythmia susceptibility (40) with a similar protocol to that used in another study also on DSP-mutant mice which yielded opposite results (37). Maybe the slight differences in the age of the animals, the details of the exercise treadmill protocol, or the different DSP mutations (deletion vs. missense) could account for part of these discrepancies which, anyhow, lay out of the scope of this review and could be clarified with future studies assessing this issue.

## Clinical Series Analyzing the Effect of Exercise on ACM

In line with the previous sections of this manuscript regarding the causes of sports-associated SCD and the results on exercised ACM animal models, also clinicians have gathered a valuable piece of evidence by analyzing clinical series of ACM patients engaged in sports. Shown in Supplementary Table 2 is a list of observational studies which have reported outcomes in clinical series.

Taken all together, a definite deleterious effect of exercise on the structural and the arrhythmic phenotype of ACM has been proved. Some studies focus their interest on sports training defined in terms of intensity and duration (either competitive or recreational), but others broaden the analysis to physical activity in general. Some interesting series have helped to define the specific risk of physical activity in mutation carriers not exhibiting an overt ACM phenotype, concluding that exercise also promotes disease progression in this scenario (41, 42). Others have assessed the effect of exercise restriction to find out an improvement in outcomes (43, 44) regardless of the previous level of training (45). Although more exercise causes more electrical and/or structural progression (41, 43, 46–48), the intensity rather than the duration of the exercise performed seems to play a more determinant role. Indeed, intensity rather than duration is strongly linked to adverse prognosis (49, 50) and, furthermore, its reduction more effectively improves the arrhythmic burden (44). Also in the scenario of ACM patients with ablation for ventricular tachycardia, exercise activity was an independent risk factor for future LAEs (51, 52). Aiming to give a quantitative recommendation to patients, several authors suggested that <2.5 h per week and <6 METS both in probands and relatives (50) or <650 MET-Hr/years (metabolic equivalent hours obtained from multiplying METs by duration) in relatives (49) could be safe and healthy. Accordingly, a precise recommendation has been introduced in current guidelines (Table 2). From an ambiguous qualitative perspective, one paper highlighted that recreational but not competitive sports may be safe in ACM patients since their practice does not aggravate prognosis in comparison to inactive patients (53). However, reports on LAEs triggered by recreational sports in ACM patients (54) and the wide range of the intensity of recreational sports hamper the extrapolation of this result to everyday practice so that sports quantification in terms of intensity and duration is preferred over a recreational vs. competitive sports classification. Finally, the hypothesis of the interaction of environmental and genetic factors on the final ACM phenotype remarkably gathers strength in this scenario. Indeed, the more weight of the environment (sports), the least influence of genetics and, vice versa; highly lethal mutations entail such a poor prognosis that there is little room left for environmental influence on the final phenotype. Thus, patients with a negative genetic result appear to have the greatest influence of sports on their structural and electric ACM phenotype (42), whereas in carriers of the dangerous TMEM43 S358L mutation, the impact of exercise on prognosis fades out by increasing the threshold up to 9 MET-Hr/day (3,285 MET-Hr/year) in order to find significant differences in LAEs (55).

**Figure 4:**
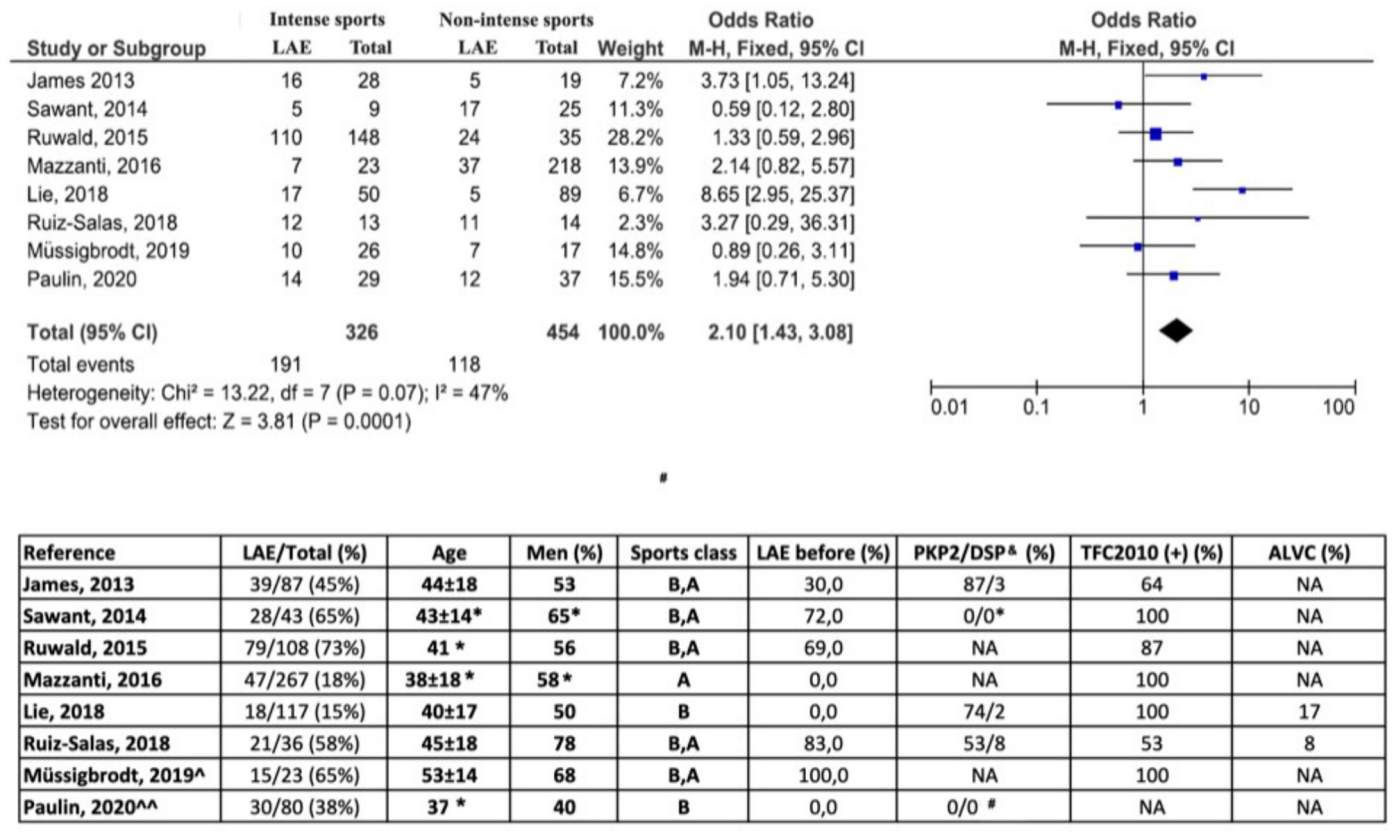
Forest plot represents standardized (5-year) rate of life-threatening arrhythmic events (LAE) and 95% CI from eight selected studies referenced in the text. Odds ratios for intense vs. non-intense exercise were calculated per study. Pooled data represented in the bottom black diamond (Review Manager 5.4). Bottom table summarizes relevant extracted demographic and clinical data from the eight selected articles. N, number; Y, years; FU, follow-up; TFC2010, Definite Task Force Criteria for arrhythmogenic right ventricular cardiomyopathy; ALVC, arrhythmogenic left ventricular cardiomyopathy; ∧VT ablation series, ∧∧ICD series, primary prophylaxis. Sports class: sports classification depending on physical activity before (B)/after (A) recruitment. ^&^Data referred to the total sample size referred at the second column, even though some patients may not have had genetic studies performed and, if done, others may not have mutation identified. ^*^Figure approximated from the data given in the paper. ^#^All carriers of the mutation TMEM43 S358L. NA, not available.

Suitable papers from Supplementary Table 2 were further processed to integrate all those series in a meta-analysis with stronger evidence. First, we acknowledge certain limitations in data collection derived from the great differences observed in the design of the studies. There were also differences in assessing the sport performed, sometimes before recruitment, others before and after, and occasionally only the sport performed after enrollment was used to classify patients. As observed in Figure 4, our pooled data confirm that ACM patients engaged in high-intensity sports activities have a significant two-fold increase in the risk of developing LAE when compared to ACM patients who report lower physical activity habits (OR 2.1, 95% CI 1.43–3.8). Unfortunately, we have not been able to compare the age of the LAEs in both groups, but, as observed in some of the reviewed papers, it was significantly younger in the sports-engaged ACM patients. Our meta-analysis approach to evaluate the risk of LAE in ACM patients depending on their sports practice is brand new and provides strong and compelling evidence to reassure the need of sports restriction in this scenario. Furthermore, our systematic review of all these papers has allowed us to highlight that the vast majority of patients exhibited a classical phenotype (definite Task Force Criteria 2010), that the representation of patients harboring non-PKP2 mutations was really small, and that the percentage of patients with left ventricular involvement remains widely unknown. Thus, clinicians should be aware that little evidence now support any exercise recommendation out of the clinical profile of the papers herein reviewed.

## Strenuous Exercise as Stand-Alone Cause of ACM

Strenuous exercise may induce cardiac adaptation in the so-called athlete heart. Further maladaptive remodeling may lead to develop features resembling ACM at one or both ventricles.

Endurance athletes are exposed to myocardial damage as a direct consequence of their high-level training with a subsequent rise in blood BNP, CK-MB, troponin-T, and troponin-I (56). Additionally, athletes exhibit an increased risk of right ventricular remodeling and ventricular arrhythmias typically arising from the right ventricle, yielding a variable percentage of individuals fulfilling ACM Task Force Criteria 2010, up to 59% definite and 30% borderline/possible by some authors (57) or 57% borderline/possible in other series (58). However, these athletes have been proved to have a lower than expected rate of mutation-positive studies (13% in athletes vs. 50% reported in papers concerning ACM in general) (59). Thus, the so-called exercise-induced ACM was proposed to explain the occasional development of a phenotype identical to classical ACM in endurance athletes (mostly cyclists) (56), sometimes with subepicardial right ventricular outflow tract scar as substrate for fast ventricular tachycardia (60), which was then reproduced in a mouse model (61). The incidence of such a cardiac behavior is rare, around 1/1,000 at risk individuals but suspected to be as high as 1/100 in top elite athletes (56). Remarkably, the continuum between sports-triggered right ventricular remodeling and an ACM phenotype, the low incidence of exercise-induced ACM, and the limitation of the yield of the genetic studies (see introduction) preclude an accurate definition of the outcomes of this entity. Thus, current guidelines on sports recommendations (11) do not make any distinction between genetic and suspected exercise-induced ACM in terms of exercise restriction once an overt ACM phenotype is present. Among the clinical series reviewed on the topic “ACM and sports/exercise,” two of them specifically focused on outcomes in gene elusive individuals (41, 46). As previously commented, this group might include both genetic ACM with negative genetic results and sports-induced ACM. Supplementary Table 2 includes a brief summary of these papers showing that, in comparison to gene-positive individuals, gene-negative patients need to train harder to develop the ACM phenotype (41) and that sports restriction reduced ventricular arrhythmias in them more often than in gene-positive patients (46).

Finally, also the left ventricle can suffer some sort of exercise-triggered damage identified as non-ischemic subepicardial scars mimicking those observed in left dominant ACM patients, healed myocarditis, or even Fabry disease. Indeed, myocardial fibrosis detected by late gadolinium enhancement resonance imaging has been reported to occur in up to 50% of asymptomatic athletes and veteran triathletes, mostly at meso/epicardial inferolateral left ventricular walls, mostly in men, and associated with higher blood pressure, myocardial mass, and longer cumulative distances (62–65). A cycling race distance of >1,880 km completed during competition had the highest accuracy to predict late gadolinium enhancement (63). On the contrary, other clinical series did not find any late gadolinium enhancement in endurance athletes (58) so that the real prevalence of this feature unfortunately remains widely unknown.

## Conclusions

Arrhythmogenic cardiomyopathy accounts for roughly 10% of all sports-related SCDs which implies a five-fold risk of suffering an SCD in comparison to that of patients with hypertrophic cardiomyopathy based on sports-related SCD series. The young age of the athletes and certain specific geographical regions may profoundly increase these estimates. Despite the beneficial effect of exercise in general population, physical activity promotes the onset and aggravates the structural and electrical features of ACM both in preclinical models and in clinical series. The very few exceptions of papers reporting conflicting results may account for differences in the design of the studies. Our pooled data based on previously published studies confirm that high-intensity sport is associated with a two-fold increase in the risk of LAE. The promising preclinical data which support a beneficial effect of drug intervention to lower preload may open avenues in the future to partially mitigate the negative impact of sports on ACM patients who, despite international recommendations, decide to maintain their high-intensity physical activities. Already reported evidence shows that the intensity rather than duration of exercise is responsible for this negative effect. Moreover, sports restriction seems to partially improve the phenotype but does not completely blur the risk of structural progression and arrhythmic events in previously trained athletes, so that the decision to implant an ICD should remain independent of their degree of sport engagement. Physical exercise at <6 METS and <2.5 h (150 min) per week could be safe for both ACM patients and mutation carriers.

**Figure 5:**
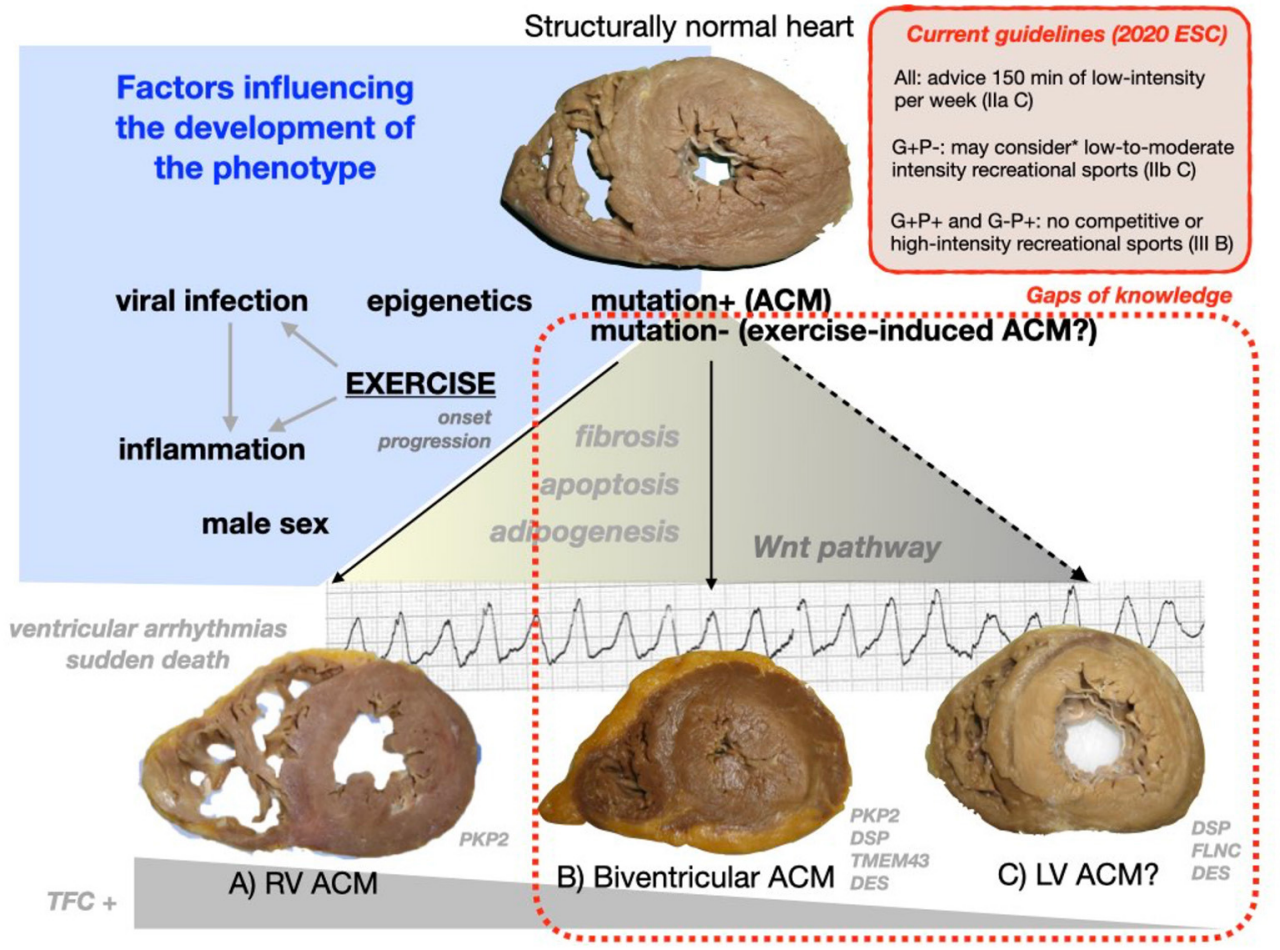
Central figure. The progression of a structurally normal heart harboring a mutation or without any genetic hit to an ACM phenotype involving just the right ventricle **(A)**, both ventricles **(B)** or the left ventricle in isolation **(C)**. **(A–C)** Were obtained at autopsies of patients with ACM and sports-triggered sudden cardiac death. Different factors may modulate this transition yielding structural and arrhythmic features through the Wnt pathway dysregulation (as presented in Table 3). The rate of patients fulfilling TFC 2010 and the specific mutated genes underlying may widely differ among ACM phenotypes A–C. The most important genotype–phenotype associations based on the authors clinical and forensic experience are shown at the bottom of this figure (further information on this topic is provided in Table 1). *Only applicable to low risk patients with no history of cardiac arrest/ventricular arrhythmias, unexplained syncope, minimal structural cardiac abnormalities, <500 PVCs/24 h, and no evidence of exercise-induced complex ventricular arrhythmias (11). ACM, arrhythmogenic cardiomyopathy; ESC, European Society of Cardiology; G, genotype; P, phenotype; TFC, Task Force Criteria 2010; LV, left ventricular; RV, right ventricular; PKP2, plakophillin-2; DSP, desmoplakin; DSG2, desmoglein-2; DES, desmin; TMEM43, transmembrane protein 43; FLNC, filamin C; PVCs, premature ventricular complexes.

Remarkably, the knowledge herein reviewed suffer from some challenging limitations (knowledge and gaps are showed in Figure 5).

First, evidence has been gathered in preclinical and clinical series with *classical* ACM phenotypes often *requiring a definite* Task Force Criteria 2010 at recruitment and with a high percentage of *plakophillin-2* mutations. The clinical characterization of left ventricular involvement was incomplete in most of the available papers and the presence of mutations in genes typically associated with left ventricular phenotype is rare (Supplementary Table 2). Therefore, the global effect of physical activity and the threshold for a safe exercise recommendation in left ventricular ACM and in carriers of non-plakophillin-2 mutations (such as desmoplakin or desmin) have not been yet specifically addressed.

Second, although it has been suggested that *strength exercise* (predominantly static) might be safer for ACM patients than sports with a high dynamic demand, it has not been specifically studied and proved so far. Sports with a low dynamic and static component include bowling, cricket, curling, golf, riflery, and yoga.

Furthermore, to *what extent endurance exercise may induce* a right ventricular or left ventricular ACM phenotypes itself or if some sort of genetic background needs to be present remains unknown.

Future research endeavors may hopefully fill in these gaps soon (Figure 2). Personalized medicine will probably take into account a wide range of genetic, epigenetic, and epidemiological variables, to accurately assist clinicians willing to recommend a safe exercise-practice to ACM patients.

## Data Availability Statement

Publicly available datasets were analyzed in this study. This data can be found here: we have reviewed the available papers on sports and arrhythmogenic cardiomyopathy to show the evidence in a systematic approach to the readers. No original dataset have been used for this article.

## Author Contributions

JM-S and JS-M reviewed the papers regarding the effect of sport on ACM in clinical series, prepared Supplementary Table 2, and edited the manuscript. PM and EZ performed the Pubmed search, reviewed the papers regarding the sports-triggered SCD, prepared Supplementary Table 1, selected the pictures included in Figures 1, 5 from her personal archive, and edited the manuscript. JG, MS-M, and AB-B reviewed the papers regarding the pre-clinical evidence of sports on ACM, prepared the Table 3, and edited the manuscript. LM-D reviewed the current knowledge about the genetic background of ACM, prepared Tables 1, 2, and edited the manuscript. JG and EZ prepared the data from the papers to perform the meta-analyses. JG and MS-M performed the statistical analyses. MS-M built the Forest plots and draw the Figures 3, 4. EZ conceived the review manuscript, supervised the tables, figures, wrote, and edited the manuscript. All authors contributed to the article and approved the submitted version.

## Funding

This work was in part supported by grants from Instituto de Salud Carlos III and FEDER Union Europea, Una forma de hacer Europa (PI18/01582, PI18/01231).

## Conflict of Interest

The authors declare that the research was conducted in the absence of any commercial or financial relationships that could be construed as a potential conflict of interest.

## Publisher's Note

All claims expressed in this article are solely those of the authors and do not necessarily represent those of their affiliated organizations, or those of the publisher, the editors and the reviewers. Any product that may be evaluated in this article, or claim that may be made by its manufacturer, is not guaranteed or endorsed by the publisher.
