# Corrigendum: Facts and Gaps in Exercise Influence on Arrhythmogenic Cardiomyopathy: New Insights From a Meta-Analysis Approach

**DOI:** 10.3389/fcvm.2021.816280

**Published:** 2022-02-04

**Authors:** Julia Martínez-Solé, María Sabater-Molina, Aitana Braza-Boïls, Juan J. Santos-Mateo, Pilar Molina, Luis Martínez-Dolz, Juan R. Gimeno, Esther Zorio

**Affiliations:** ^1^Cardiology Department, Hospital Universitario y Politécnico La Fe, Valencia, Spain; ^2^Laboratorio de Cardiogenética, Unidad de Cardiopatías Familiares, Instituto Murciano de Investigación Biosanitaria (IMIB-Arrixaca), Murcia, Spain; ^3^Unidad CSUR (Centros, Servicios y Unidades de Referencia) en Cardiopatías Familiares, Hospital Universitario Virgen de la Arrixaca, Murcia, Spain; ^4^CIBERCV, Center for Biomedical Network Research on Cardiovascular Diseases, Madrid, Spain; ^5^Unidad de Cardiopatías Familiares, Muerte Súbita y Mecanismos de Enfermedad (CaFaMuSMe), Instituto de Investigación Sanitaria La Fe, Valencia, Spain; ^6^Cardiology Department, Hospital Universitario Virgen de la Arrixaca, Murcia, Spain; ^7^Instituto de Medicina Legal y Ciencias Forenses de Valencia, Histology Unit, Universitat de València, Valencia, Spain

**Keywords:** sports, exercise, arrhythmogenic cardiomyopathy, disease progression, risk factors

In the original article, there was a mistake in Figure 3 as published. During the submission process the upload of our original Figure 3 repeatedly failed due to the size of the ppt file. Due to an exportation error, the new Figure 3 changed its x axis from increases 10 by 10 to increases 16 by 16 approximately and the error bars were not adjusted accordingly. The corrected Figure 3 appears below.

**Figure 3:**
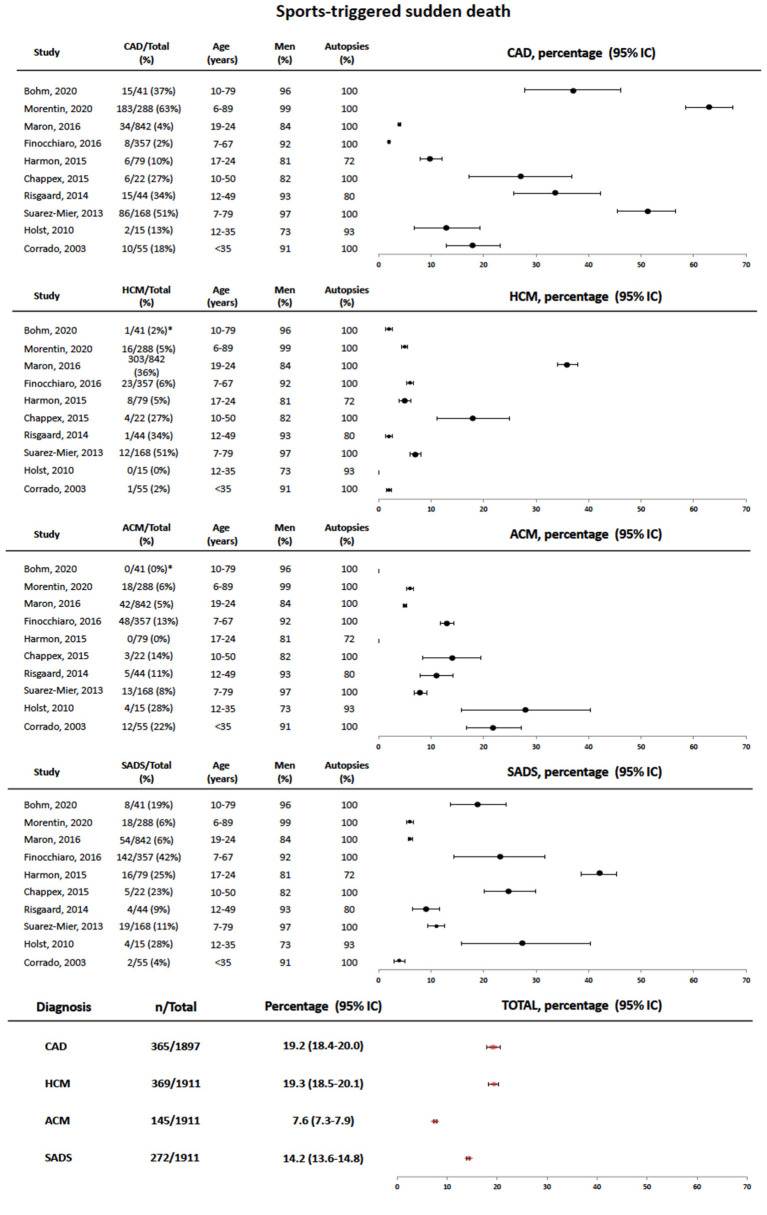
Forest plot showing pooled data of the causes of sports-triggered sudden cardiac death in different series referenced in the text. At the bottom, total estimates are provided for each diagnosis. SCD, sudden cardiac death; CAD, coronary artery disease; SADS, sudden arrhythmic death syndrome; HCM, hypertrophic cardiomyopathy; ACM, arrhythmogenic cardiomyopathy. ^*^The causes of death can be only retrieved form the 41 cases of sports-related sudden death cases with autopsy.

The authors apologize for this error and state that this does not change the scientific conclusions of the article in any way. The original article has been updated.

## Publisher's Note

All claims expressed in this article are solely those of the authors and do not necessarily represent those of their affiliated organizations, or those of the publisher, the editors and the reviewers. Any product that may be evaluated in this article, or claim that may be made by its manufacturer, is not guaranteed or endorsed by the publisher.

