# Supplementary material for: Facts and Gaps in Exercise Influence on Arrhythmogenic Cardiomyopathy: New Insights From a Meta-Analysis Approach
Source: Front Cardiovasc Med. 2021 Oct 18;8:702560. doi: 10.3389/fcvm.2021.702560 (PMC8558346; doi:10.3389/fcvm.2021.702560)
Supplement: Supplementary file 1 [file Table_1.docx]

Online Material Table 1. Sports-triggered sudden cardiac death series reporting the cause of death

|  | **N** | **Years of study** | **Location, source**  **Autopsies (%)** | **F%**  **M/F** | **Mean age**  **(range)**  **years old** | **Sports** | **Causes** |
| --- | --- | --- | --- | --- | --- | --- | --- |
| **Corrado, 2003**  **(ref 20)** | 55* | 21  *(1979-1999)* | Postmortem investigation of SCD in the young in Veneto (Italy).  100% autopsies. | 9%  *(50/5)* | 23±7  *(<35)* | Soccer  Basketball  Swimming | ACM 22%  CAD 18%  Coronary anomalies 13%  Myocarditis 9%  HCM, DCM and Aortic Rupture each 2%  SADS 4% |
| **Holst, 2010 (21)** | 15 | 7  *(2000-2006)* | Danish Cause of Death Registry, autopsy results and medical records (Denmark).  93% autopsies. | 27%  *(11/4)* | 26  *(12-35)* | Running  Soccer  Handball  Cycling  Aerobics  Boxing | ACM 28%  SADS 28%  CAD 13%  Myocarditis, Coronary anomalies and Sarcoidosis each 7% |
| **Suarez-Mier, 2013**  **(ref 22)** | 168 | 10  *(1995-2010)* | Forensic postmortem investigation of sports-related SCD (Spain).  100% autopsies. | 3%  *(163/5)* | 37±16  *(9-79)* | Cycling  Soccer  Running  Gymnastics | CAD 51%  SADS 11%  ACM 8%  HCM 7% |
| **Risgaard, 2014**  **(ref 23)** | 44 | 3  *(2007-2009)* | The Danish Cause of Death Registry (Denmark).  80% autopsies. | 7%  *(41/3)* | 41±10  *(12-49)* | Running  Cycling | CAD 34%  ACM 11%  HCM 2%  Hypertrophic heart 9%  SADS 9% |
| **Harmon, 2015 (ref 24)** | 79 | 10  *(2003-2013)* | The National Collegiate Athletic Association database (USA).  72% autopsies | 19%  *(64/15)* | (17-24) | Basketball  Soccer  Football | SADS 25%  Coronary anomalies 11%  Myocarditis 10%  CAD 10%  HCM 5%  Idiopathic LVH 5% |
| **Chappex, 2015**  **(ref 25)** | 22 | 10  *(1995-2010)* | Forensic postmortem investigation of sports-related SCD in Laussane (Switzerland).  100% autopsies. | 18%  *(18/4)* | 37±10  *(10-50)* | Hiking  Swimming | CAD 27%  SADS 23%  HCM 18%  ACM 14%  Myocarditis 9% |
| **Finocchiaro, 2016 (ref 26)** | 357 | 20 *1994-2014* | Regular sport-triggered SCD cases referred to the CRY Centre at the Royal Brompton Hospital (UK).  100% autopsies. | 8%  *(330/27)* | 29±11  *(7-67)* | Running Cycling  Gymnastics  Swimming  Weightlifting | SADS 42%  Idiopathic LVH and/or fibrosis 16%  ACM 13%  HCM 6%  CAD 2% |
| **Maron, 2016**  **(ref 27)** | 842 | 32  *(1980-2011)* | The US National Registry of Sudden Death in Athletes (USA).  100% autopsies. | 16%  *(54/10)* | -  *(19-24)* | Basketball  Football  Soccer  Running  Swimming | HCM 36%  Coronary anomalies 19%  Myocarditis 7%  ACM 5%  CAD and MVP each 4%  Aortic Rupture 3%  DCM and LQTS each 2%  WPW 1% |
| **Morentin, 2020**  **(ref 28)** | 288 | 8  *(2010-2017)* | Forensic postmortem investigation of sports-related SCD (Spain).  100% autopsies. | 1%  *(284/4)* | 44±14  *(6-80)* | Gymnastics  Cycling  Running  Soccer  Swimming | CAD 63%  SADS 6%  ACM 6%  HCM 5%  Coronary anomalies 2%  Aortic dissection 1% |
| **Bohm, 2020**  **(ref 29)** | 240 | 6  *(2012-2018)* | Prospective Heart Arrest in Sports Registry (Germany).  17% autopsies. | NA | *(10-79)* | Soccer  Running  Swimming  Cycling | Data available only from the 41 SD with autopsy:  CAD 37%  Myocarditis 22%  HCM, DCM and sarcoidosis each 2%  SADS 19% |

*11% were sudden deaths in athletes not related to exercise. SCD: Sudden cardiac death. CAD: coronary artery disease. SADS: Sudden arrhythmic death syndrome. HCM: Hypertrophic cardiomyopathy. ACM: Arrhythmogenic cardiomyopathy. DCM: dilated cardiomyopathy. LQTS: long QT syndrome. MVP: mitral valve prolapse. WPW: Wolff-Parkinson-White syndrome. LVH: Left ventricular hypertrophy. CRY: Cardiac Risk in the Young. NA: not available
