# Supplementary material for: Facts and Gaps in Exercise Influence on Arrhythmogenic Cardiomyopathy: New Insights From a Meta-Analysis Approach
Source: Front Cardiovasc Med. 2021 Oct 18;8:702560. doi: 10.3389/fcvm.2021.702560 (PMC8558346; doi:10.3389/fcvm.2021.702560)
Supplement: Supplementary file 2 [file Table_2.docx]

Online Material Table 2. Summary of papers focussed on the effect of sports on ACM patients.

|  | **Clinical ang genetic characteristics** | **Sports assessment and definitions** | **Results** |
| --- | --- | --- | --- |
| **James, 2013**  **(ref 43)** | N=87 (41 probands, 46 relatives).  Men 53%.  Mean age 44±18 years.  All desmosomal mutation carriers (87% PKP2, 3% DSP).  64% definite TFC 2010.  No mention to LV involvement. | Interviews.  Endurance athletics: high energetic demand >70% max O2, at least 50 h/year at vigorous intensity. | Endurance athletes (binary analysis) and those at the highest quartiles (quantitative analysis) were associated to a more severe phenotype (more HF symptoms and at a younger age), a higher rate of definite TFC 2010 and more electrical burden (shorter VA-free survival). Those patients at the highest quartile who reduced exercise, had lower rates of VA during follow up. |
| **Sawant, 2014**  **(ref 42)** | N=82 (all probands).  Men 65%.  Mean age 43±14 years.  39 (48%) carriers of desmosomal mutations (77% PKP2, 5% DSP).  43 (52%) had a negative genetic study (G-).  100% definite TFC 2010.  No mention to LV involvement. | Interviews.  Endurance athletics: as above. | G- patients had a greater environmental influence than G+ patients since they were more often endurance athletes, had practiced more vigorous exercise and reported a lower rate of familial disease. Moreover, those G- at the highest quartile of sports before diagnosis, were more likely to fulfill TFC 2010 criteria, were younger and had a shorter VA-free survival. |
| **Saberniak, 2014 (ref 41)** | N=110 (65 probands, 45 relatives).  Men 58%.  Mean age 42±17 years.  75% carriers of desmosomal mutations (91% PKP2, 3% DSP).  71% definite TFC 2010.  LVEF 54±8.  No mention to LV LGE. | Interviews.  Athlete: physical activity with intensity ≥6 METs for ≥4 hours/week (≥1440 METs x min/week) during minimum 6 years. | Athletes showed reduced biventricular function compared with non-athletes in ACM patients and in mutation-positive family members. The amount and intensity of exercise activity was associated with impaired LV and RV function. Exercise may aggravate and accelerate myocardial dysfunction in ACM. |
| **Sawant, 2016**  **(ref 49)** | N=37 (9 probands, 28 relatives).  Men 46%.  Mean age 40±17 years.  100% carriers of a heterozygous radical PKP2 mutation.  60% definite TFC 2010.  No mention to LV involvement. | Interviews.  Endurance athletics: as above. | Endurance exercise was associated with definite TFC 2010 and VA whereas exercise restriction to the opposite. Affected relatives accumulated 2.8-3.5-fold greater MET-Hr exercise than the AHA-recommended minimum (650 MET-Hr/year). Thus, exercise within the upper bound of the recommended minimum may be reasonable for unaffected mutation carriers. |
| **Ruwald, 2015 (ref 53)** | N=108 (all probands).  Men 56%.  Mean age, roughly 41years.  100% definite/borderline TFC 2010.  No data regarding genetic background.  No mention to LV involvement. | Interviews.  Patients: inactive, recreational or competitive.  Sports: high dynamic or low to moderate dynamic. | Competitive sports were associated with a significantly higher risk (2-fold) of VA/death and symptoms at a younger age. When compared with inactive patients, recreational sport was not associated with earlier onset of symptoms or increased risk of VA/death. |
| **Mazzanti, 2016 (ref 51)** | N=301 (163 probands, 138 relatives).  Men 58%.  Mean age 38±18 years.  38% carriers of desmosomal mutations (mostly PKP2, rare DSP, DSG2 and DSC2) with incomplete studies in 6-12% probands.  Follow-up 5.8 years.  100% definite TFC 2010.  LVEF 61±8%.  LGE on LV or RV or both 31%. | Questionnaires.  Strenuous exercise: Regular (≥5 hours/week for ≥1 year) participation in vigorous-intensity physical activities (≥6 METS). | Life-threatening arrhythmic events (LAE) were registered in 1.5-4/100 person-years between 0-60 years (peak at 21-40 years) with a cumulative probability of a first LAE of 30% at 15 years of follow-up. Best predictors were: atrial fibrillation, syncope, strenuous exercise, hemodynamically tolerated sustained monomorphic ventricular tachycardia, and male sex. Anti-arrhythmic treatment did not modify the risk of first and recurrent LAEs. |
| **Wang, 2018 (ref 44)** | N=119 (107 probands, 12 relatives).  Men 60%.  Mean age 34±15 years.  100% definite/borderline TFC 2010 and ICD carriers (36% primary prevention).  41% had a negative genetic study (G-).  Follow-up 5.1 years.  LVEF<45% 17%.  No mention to LV LGE. | Interviews.  Endurance athletics: as above.  Exercise change: annual exercise duration and dose in the 3 years before clinical presentation minus that after presentation. | 74% of patients reduced exercise dose and 66% suffered appropriate ICD therapies. Reduction in exercise dose (in comparison to duration reduction) conferred greater reduction in VA. G- patients and those with primary prevention ICDs benefited the most. However, 58% of athletes who reduced exercise dose by > 80% still experienced VA. Exercise reduction is unlikely to reduce VA enough in high-risk patients to alter decisions about ICD implantation. |
| **Ruiz Salas, 2018 (ref 46)** | N= 36 (all probands)  Men 78%  Mean age 45 ± 18 years.  100% definite TFC 2010 high risk*.  75% desmosomal mutation carriers (70% PKP2, 11% DSP).  Mean LVEF 57.8 ± 9.6%, 5.6% ≤35%.  17% LV involvement, 17% LV LGE . | Interviews. | Both, the appearance of VA/heart arrest and the occurrence of severe RV systolic dysfunction were inversely related to the energetic demand of the physical activity performed. |
| **Lie, 2018 (ref 47)** | N= 117 (23 probands, 94 relatives)  Men 50%.  Mean age 40 ± 17 years.  56% definite TFC 2010 without VA.  Follow up 4.2 years.  83% mutation carriers (90% PKP2, 3% DSP).  LVEF 57 ± 7%.  LGE (not specified RV and/or LV) 13%. | Interviews.  High intensity exercise: >6 METS for at least 3 years before inclusion. | The 1-, 2-, and 5-year incidence for life-threatening VA was 6%, 9%, and 22%, respectively. History of high-intensity exercise, T-wave inversions ≥V3, and greater LV mechanical dispersion were the strongest risk markers. Patients without any of these risk factors had minimal risk, whereas ≥2 risk factors increased the risk dramatically. |
| **Lie, 2018 (ref 50)** | N=173 (91 probands, 82 relatives).  Men 56%.  Mean age 41 ± 16 years.  Probands fulfilled TFC 2010.  Genetic analyses were performed  in 170 (98%) patients (64% PKP2, 5% DSP, 4% DSG2, 1% DSC2, 26% G-).  LVEF 54-58%.  No mention to LV LGE. | Interviews.  Exercise: physical activity performed on a regular basis during the past 3 year. | More prevalent VA in patients performing exercise of greater intensity (>6 METS) and duration (>2.5 hours/week). However, on multivariate analysis, intensity rather than duration was associated to VA. |
| **Lin, 2019 (ref 52)** | N=91 (all probands)  Men 52%.  Mean age 47±13 years.  Follow-up 32±26 months.  27%mutation carriers (11% DSP, 4% PKP2, 5% DSG2, 3% JUP and 3% TMEM43).  100% definite TFC 2010 with ≥1 RV VA ablation (89% success, 8.8% partial success).  Mean LVEF 56%.  No mention to LV LGE. | Interviews.  Patients: endurance athletes and non-athletes as defined by the 36th Bethesda Conference Classification of Sports. | Up to 38% suffered recurrent VA and 31% needed multiple procedures. Newly-developed circuits owing to the scar progression were observed in 72.9%of recurrent VA. The patients with repeated procedures had worsening right ventricular remodeling. The multivariate analysis revealed that history as endurance athlete significantly predicted the need to repeat procedures despite the initially successful endocardial/epicardial ablation and negative inducibility. |
| **Catto, 2019 (ref 54)** | N=2 (both probands)  Men 100%.  Aged 20 and 21 years.  100% disqualified elite athletes who decided to continue sports practice.  1 PKP2, 1 G-.  100% carriers of s-ICD  100% definite TFC 2010  Both biventricular involvement with LV dilatation, 1 with LV LGE | - | Between 3 and 12 months after ICD implantation they suffered appropriate successful therapies while practicing recreational basketball and cycling. Sports restriction should be recommended. |
| **Müssigbrodt, 2019 (ref 45)** | N=38  Men 68%.  53±14years  79% sports-engaged before ablation.  100% definite TFC 2010.  100% recruited at the ablation of ventricular tachycardias (92% success).  Follow-up 52±31 months after the procedure  No data regarding genetic background.  Mean LVEF 58%.  No mention to LV LGE. | Intra-hospital data system and by interviews.  Patients: sedentary, recreational or competitive sports (highly dynamic sports and low to moderately dynamic sports). | None continued practicing competitive sports after the procedure. Up to 39% of patients suffered ventricular tachycardia recurrences which were only associated to increased age, not to previous practice of recreational sports (at any intensity). |
| **Paulin, 2020**  **(ref 55)** | N=80 (41 probands, 46 relatives).  Men 40%.  Mean age roughly 37 years.  All carriers of the TMEM43 S358L.  No mention to TFC 2010.  LV dilatation in 56%.  No mention to LGE. | Questionnaire. | Exercise ≥9.0 MET-hours/day (high level) in the year before ICD implantation carried an adjusted 9.1-fold increased hazard of first appropriate ICD discharge (there were no deaths) relative to physical activity <9.0 MET-hours/day (moderate level). The median age from birth to first appropriate ICD therapy was 58.5 years (moderate) vs 35.8 years (high-level exercise groups). |
| **Costa, 2020 (ref 48)** | N=9 (4 proband, 5 relatives)  Men 56%.  Age 35±16y.  100% mutation carriers (5 PKP2 and 4 DSG2).  78% definite TFC 2010.  No mention to LVEF and LV LGE | - | A wide range of phenotypes (no disease, RV ACM, biventricular ACM), age at presentation and electrical stability was observed. Physical exercise was the common denominator in provoking an arrhythmic phenotype in these families. |

*High risk: VA, severe RV or LV dysfunction, and ICD carriers (36% primary prevention). G+: Gene positive. G-: Gene negative. ACM: Arrhythmogenic cardiomyopathy. HF: heart failure. VT: Ventricular tachycardia. VA: ventricular arrhythmias. ICD: implantable cardioverter-defibrillator. TFC: Task Force Criteria 2010. LV: left ventricular. RV: Right ventricular. LVEF: left ventricular ejection fraction. LGE: late gadolinium enhancement. PKP2: Plakophillin-2. DSP: Desmoplakin. DSG2: Desmoglein-2. JUP: plakoglobin. TMEM43: Transmembrane protein 43.
